# Welfare considerations during a conservation translocation of white-clawed crayfish (*Austropotamobius pallipes*): Proof of concept

**DOI:** 10.1017/awf.2026.10080

**Published:** 2026-04-13

**Authors:** Imogen Crowe, Oliver Heley, Alice Dancer, Ricardo Lemos de Figueiredo, Nicola Cooke, Jen Nightingale, Sarah Richdon

**Affiliations:** Bristol Zoological Society, Bristol, UK

**Keywords:** Animal welfare, conservation, decapod crustacean, invertebrate welfare, reintroduction, supplementation, welfare indicators

## Abstract

Translocations are a conservation method used to establish or supplement self-sustaining populations. However, research regarding the welfare implications of this strategy is limited, particularly in recipient wild populations. Introductions of captive-born individuals are commonly used to supplement populations of endangered white-clawed crayfish (*Austropotamobius pallipes*), the UK’s only native crayfish species. To determine effects of translocation, both introduced and wild crayfish were marked and PIT-tagged, then monitored through behavioural observations and body condition scoring. We undertook analysis on welfare indicators, comparing resident and captive crayfish, as well as resident crayfish pre- and post-introduction. Our results provide some evidence that translocation events alter behaviour of resident crayfish. We also found some evidence that wild and captive-born crayfish differ in behaviour, with potentially negative welfare implications. By contrast, body condition analysis showed no variation pre- and post-introduction, suggesting that captive-born crayfish can effectively adapt to natural environments. Some behavioural differences were also better explained by other predictors rather than origin of animal. Multiple influences on the behaviour of crayfish were identified, including sex and weather conditions. Moreover, we identified factors which could enhance the welfare of this species in captivity and increase translocation efficacy, such as replicating features of natural environments in hatcheries. We also establish a basis for future research which could enhance conservation and understanding of the species.

## Introduction

With growing human populations around the world, natural environments are facing increasing pressure from anthropogenic expansion and exploitation. According to the International Union for Conservation of Nature and Natural Resources (IUCN), approximately 28% of the 163,000 assessed species are threatened with extinction (IUCN 2024). Habitat degradation, urbanisation, pollution, climate change and invasive non-native species (INNS) are the main drivers of global biodiversity declines (Butchart *et al.* 2010; Pimm *et al.* 2014; Díaz *et al.* 2019). Evidence suggests that current extinction rates vastly exceed natural background rates (Ceballos *et al.* 2015), highlighting the need for successful conservation interventions.

Conservation translocations, i.e. the human-mediated movement of live organisms from one area to another, with the goal of establishing, or enhancing, self-sustaining populations (IUCN/SSC 2013; Jourdan *et al.* 2019), are a commonly used tool in conservation biology. Translocations include reintroductions (movement of individuals to areas in which a species has been expatriated), introductions (to new areas where a species was not previously recorded) and supplementations (to areas in which a population already exists) (IUCN/SSC 2013), all of which may involve the movement of animals taken from the wild or those bred or reared in captivity.

Due to increasing global habitat degradation and fragmentation, species translocations are likely to be an essential conservation strategy in the future, particularly for species with limited dispersal abilities (Jourdan *et al.* 2019). Despite their popularity, the efficacy of translocations has been drawn into question due to variability in success rates and potential costs (Pérez *et al.* 2012; Berger-Tal *et al.* 2020). The survival, viability and reproduction rates of the translocated population are often used as indicators of success in translocation programmes, but the welfare implications of these programmes for both translocated and wild populations have often been overlooked (Harrington *et al.* 2013). Welfare is intrinsically linked to translocation success and negative welfare states that may occur due to translocation can incur negative consequences for animal survival (Swaisgood 2010; Harrington *et al.* 2013), such as stress-induced mortality (Thompson *et al.* 2023). Therefore, welfare should be an important consideration in the planning and execution of animal translocations to maximise the chance of success. The *IUCN Guidelines for Reintroductions and Other Conservation Translocations* (IUCN/SSC 2013) suggests that although every effort should be made to adhere to internationally accepted standards of welfare, translocations may incur some negative welfare consequences. Stress, for example, is considered an inevitable consequence of translocations (Teixeira *et al.* 2007; Dickens *et al.* 2010; Gelling *et al.* 2012; Lèche *et al.* 2016). Acute stressors like capture, captivity, transportation, and exposure to a new environment may contribute to a state of chronic stress, which in turn affects behaviour and compromises the well-being of translocated individuals (Dickens *et al.* 2010). Additionally, translocations may disrupt established social structures, particularly if social groups are separated during the translocation process, which may have negative consequences for adaptation and survival (Shier & Swaisgood 2012; Franks *et al.* 2020). Captive breeding of animals for release also presents its own challenges, as captivity has been shown to elicit behavioural variations over generations (McPhee 2004; McDougall *et al.* 2006; May *et al.* 2016). This variation might have implications not only for the released individuals (Grueber *et al.* 2017), but also for wild populations which they may encounter (Champignon *et al.* 2012), particularly if behaviours do not align with that of their wild counterparts. The welfare impact of translocations on resident populations is notably understudied (Champignon *et al.* 2012) and, in the case of social species, the implications of introducing new individuals may be significant.

The white-clawed crayfish (*Austropotamobius pallipes* Lereboullet 1858) is one of the largest indigenous freshwater invertebrates, and the only native crayfish species in the UK (Holdich *et al.* 2009a; Nightingale *et al.* 2017). Once widespread across Europe, the species has become the focus of numerous conservation initiatives (Kozák *et al.* 2011; Nightingale *et al.* 2017) following a suspected decline of between 50 to 80% (Füreder *et al.* 2010). White-clawed crayfish are currently classed as Endangered by the IUCN (Füreder *et al.* 2010) and their decline has been largely attributed to the introduction, and subsequent proliferation, of invasive crayfish species, such as the American signal crayfish (*Pacifastacus leniusculus* Dana 1852) (Holdich *et al.* 2009b; Soto *et al.* 2023). As well as competing for resources, American signal crayfish threaten the survival of white-clawed crayfish through the vectoring of crayfish plague, caused by the oomycete (*Aphanomyces astaci* Schikora 1907), outbreaks of which can result in 100% mortality in a matter of days (Holdich *et al.* 2009b; Martínez-Ríos *et al.* 2022). Numerous translocation projects have centred upon white-clawed crayfish, with Bristol Zoological Society alone releasing over 3,000 captive-born individuals to date (Bristol Zoological Society 2024), following IUCN conservation translocation guidelines (IUCN/SSC 2013). With around 32% of all freshwater crayfish species threatened with extinction (Richman *et al.* 2015), translocation projects are likely to become more common in the future. It is important, therefore, to fully understand the implications of such projects for the translocated individuals, as well as the wild individuals they may encounter following release. For this reason, our study aimed to address the following questions:

- Do wild and captive-born crayfish exhibit behavioural differences?
- Does translocation of captive-born crayfish affect their welfare?
- Does release of captive-born crayfish affect resident crayfish welfare?
- What other factors are influential on crayfish behaviour and welfare?

Due to the complex hierarchical social structures exhibited by white-clawed crayfish (Goessmann *et al.* 2000), we hypothesise that introduction of new individuals has the potential to impact upon the welfare of a recipient population through social disturbance and increased competition. We further hypothesise that captive rearing may induce negative welfare implications for released individuals when faced with a novel environment. Here, we assess the welfare implications of translocations of captive-born, white-clawed crayfish (henceforth captive) on the released and recipient population using behavioural indices (refuge use, foraging and agonistic interactions) and body condition scores. Moreover, multiple environmental influences on crayfish species behaviour have been identified (e.g. Danêk *et al.* 2018; Ferderer *et al.* 2022). We therefore hypothesise that several environmental factors could be significant in influencing crayfish behaviour, and these may offer an alternative explanation of behaviours observed in a translocation context.

## Materials and methods

### Ethical approval

Ethical approval for the project was granted by Bristol Zoological Society, reference number BZS_2024_017 and Wild Animal Initiative, reference number SG23-022.

### Study sites

#### Bishop Sutton, Somerset, UK

**Figure 1.**
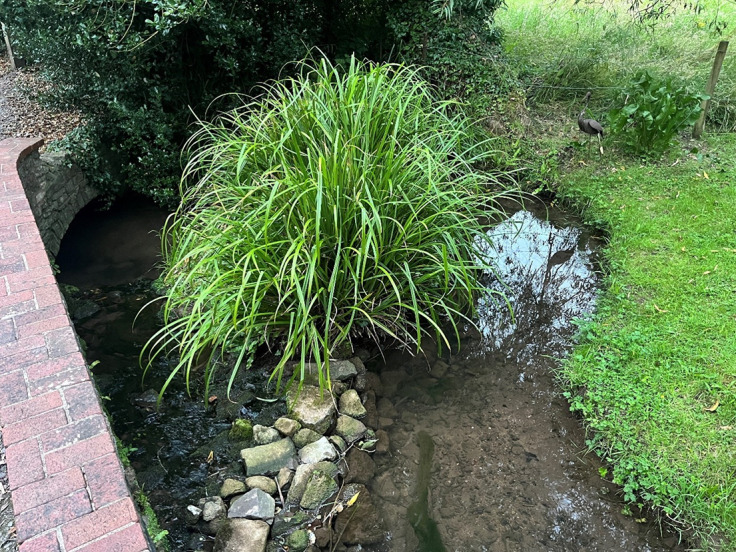
Hollow brook study site in Bishop Sutton, Somerset, UK. The stream supports a naturally occurring population of white-clawed crayfish (*Austropotamobius pallipes*).

The wild site stream bed largely consists of fine silt and medium to large rocks, with a deep undercut on each side of the bank which the crayfish use for refuge (Figure 1). The water is usually slow moving but is prone to spates in high rainfall events. The shallow water allowed good visibility of the wild population during behavioural data collection.

#### Bristol Zoo Gardens Hatchery, Bristol, UK

Captive study individuals were isolated within an outdoor, shaded black polyurethane holding tank (400 L), on a closed recirculating system. During behavioural survey periods, the mesh tank lid was lifted allowing visibility of crayfish and exposure to environmental features, such as weather and lunar phase. Water was mechanically and biologically filtered, then UV-treated before recirculating and water changes of approximately 25% occurred weekly. Temperature was controlled using water chillers to mimic *in situ* water temperature. No additional lighting was provided to the tank outside of survey hours. Substrate in the tank consisted of aquarium gravel and crayfish had access to two types of artificial refuge, bricks or short sections of PVC pipe which were glued together with aquatic-safe silicone. Feedings occurred daily and crayfish were fed a diet of frozen bloodworm, mysis, brine shrimp (Aquadip BV, Oss, The Netherlands), and a shredded vegetable mix containing carrot, cabbage, seaweed and spirulina (a nutrient-dense blue green algae).

### Study individuals

A wild resident population of white-clawed crayfish was observed at the study site in Bishop Sutton. The population was naturally occurring and of mixed sex and age.

Captive-born, white-clawed crayfish were observed pre- and post-release. The study individuals were hatched in 2022 (two years of age, approximately) from wild-caught berried females, and reared in captivity at Bristol Zoo Gardens, under licence. Ten individuals were available for the study, however due to mortality within the population, only six individuals survived to be released into the wild. Cause of death was undetermined but was not believed to be related to tagging as most mortality occurred several weeks post-tagging. Study animals were derived from the wild population in Bishop Sutton and reintroduced to the same area to maintain the genetic integrity of the population, however this limited the number of individuals available for our study.

### Trapping, tagging, marking and body condition assessment

**Figure 2.**
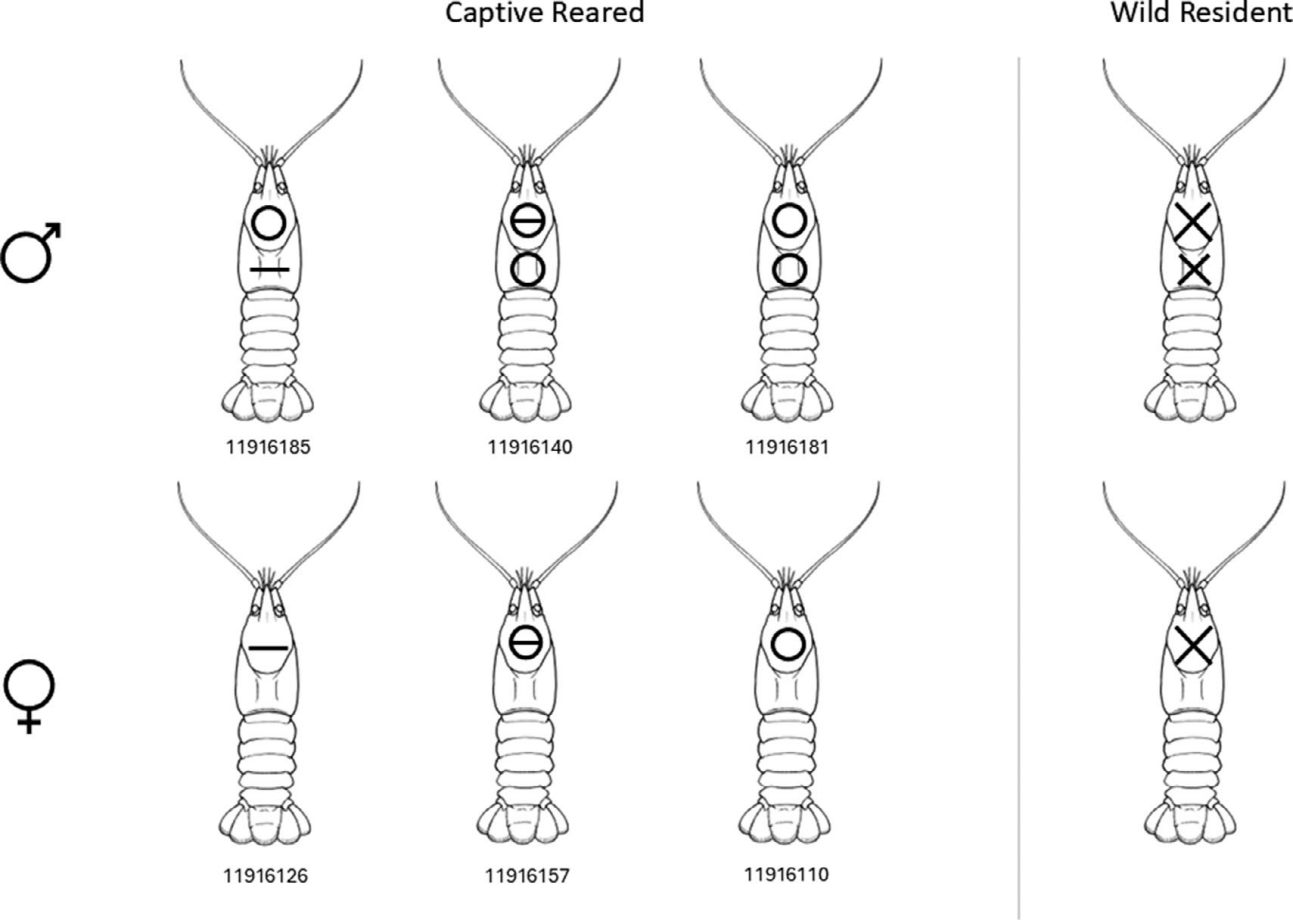
Marking patterns for captive-reared and wild resident white-clawed crayfish (*Austropotamobius pallipes*). Crayfish were marked using a white waterproof paint marker. Six released crayfish were individually marked to enable identification during behavioural observations. Wild resident individuals (n = 116) were differentiated by sex.

Prior to data collection, twenty-six artificial refuge traps were deployed in the stream on 26 June 2024, followed by 15 baited traps, deployed on 2 July 2024. All traps were checked and removed at 0900h on 3 July 2024. A total of 116 wild individuals (50 males and 66 females) were caught, which were tagged and marked, then returned to the stream. Captive-born individuals (three male and three female) were marked and tagged on 1 July 2024. A permanent waterproof paint-marker (Dykem® BRITE-MARK®, ITW Dymon, Olathe, USA) was used to mark the carapace of the captive-born and wild individuals to differentiate the sex and the provenance of the individuals during behavioural monitoring (Figure 2). Captive animals were marked individually to enable sex and individual identification. Individuals were marked on the carapace to minimise the potential for marks to be lost. Marks on chelae were avoided as crayfish may lose chelae through aggressive interactions with conspecifics (Berber *et al.* 2024). The Dykem marker is xylene free and was selected due to its use in previous decapod crustacean studies where it was shown to be safe and provide a durable mark (Ramalho *et al.* 2010; McFarlane *et al.* 2019a,b). Despite this, some individuals lost marks over the course of the study due to moulting or wear. Moreover, not all wild crayfish could be marked during trapping, meaning some wild individuals’ sex could not be determined during behavioural surveys. All captive-born and wild resident individuals were tagged using passive integrated transponder (PIT) tags, to enable individual identification. Tags measuring 7 mm × 1.35 (length × width; Loligo Systems, Viborg, Denmark) were inserted into the abdominal muscle of the third ventral abdominal section of the crayfish using a 1.78-mm-gauge hypodermic needle, as described in Nightingale *et al.* (2018a). The tag codes were recorded using a APR250 PIT tag reader (Agrident GmbH, Barsinghausen, Germany) along with the carapace length and body condition of each individual. Body condition was recorded as binomial data. A score of 0 or 1 was allocated across 15 categories, with 0 indicating perfect condition and 1 indicating compromised condition; from this, each received a total score out of 15 (Table S1; Supplementary material). Previous studies have shown this tagging technique does not significantly affect survival, growth or fecundity in crayfish with a carapace length greater than 24 mm (Nightingale *et al.* 2018a). This technique has since been used on smaller individuals (> 21 mm) and shown no adverse effects (J Nightingale, personal communication 2024).

### Study period

Behavioural data collection commenced on 1 July 2024 at the captive site and 13 July 2024 at the wild site and continued until 18 September 2024. Captive-born crayfish were released into the stream on 12 August 2024. Trapping and body condition monitoring occurred at the start (3 July 2024) and end (18 September 2024) of the survey period.

### Environmental parameter data collection

Throughout the study, water quality was monitored at both study sites (captive zoo site and wild stream site). Ammonia (NH_3_), nitrate (NO_2_), nitrite (NO_3_) and calcium (Ca) were tested using Salifert® Profi test aquarium test kits (Salifert®, Duiven, The Netherlands) and phosphate (PO_4_), pH and alkalinity (KH) were tested using Palintest reagents (AP177 Phosphate LR, AP130 pH Phenol Red, AP188 Alkaphot) and Palintest Lumiso Expert photometer (Halma PLC, Buckinghamshire, UK). Dissolved oxygen was also measured weekly, *in situ*, using Lutron Electronics PDO-519 Pen-type, Auto- calibration dissolved oxygen metre (Lutron Electronic Enterprise Co Ltd, Taipei, Taiwan). Stream and hatchery temperature was monitored continuously using TinyTag Aquatic 2 TG-4100 data loggers (Gemini Data Loggers Ltd, West Sussex, UK). Other environmental parameters, including lunar phase, ambient temperature and Met Office weather conditions (Met Office undated), were recorded at the start of each survey. Temperatures ranged from 10.55–16.59°C over the course of the behavioural study period. Potential predators were observed within the wild study site, including brown trout (*Salmo trutta* Linnaeus 1758) and brown rat (*Rattus norvegicus* Berkenhout 1769), observations that were also included at the start of each behavioural observation.

### Behavioural data collection

Behaviour data were collected using ZooMonitor (version 4.1) (Lincoln Park Zoo 2022), an app used to create ethograms and record behaviour durations. An ethogram was developed prior to data collection (Table 1) to capture the full behavioural repertoire of white-clawed crayfish. Initial stages of ethogram development consisted of comparing and compiling several ethograms from previous studies (Bergman & Moore 2003; Panksepp & Huber 2004; McFarlane *et al.* 2019b). From these studies, welfare-specific behaviours were selected, with some reference to Narshi *et al.* (2022). The ethogram was piloted on captive individuals, and necessary changes were made. Observations began once the first animal visible was spotted each evening, and the time of day of emergence was recorded. The number of visible active individuals was recorded at the start of each observation period. From these, a focal individual was selected at random using a random number generator and counting individuals from the left to right of each study site. Where possible, marked individuals were preferentially sampled. Continuous sampling was carried out on the focal individual in 30-min durations. In instances where focal individuals left the area visible to surveyors, behaviour was recorded under ‘no visible behaviour’. In infrequent instances where crayfish did not return after 3 min, the survey was terminated and a new focal individual randomly selected for survey; terminated surveys were not included in analysis. Data were collected by two observers throughout the study. Every month of observations, both observers recorded the same individual to test interobserver reliability, data were omitted from analysis. Baseline data were collected on both captive and wild populations pre-release. The combined population was subsequently monitored post-release, following the same method. Data collection took place between 2030 and 0030h at both sites, using red head torches to minimise disturbance to crayfish (Kennedy & Bruno 1961; Cheng *et al.* 2023). Prior to the start of each survey period, crayfish were allowed to acclimate to the light source for a period of 10 min. Emergence times were recorded for both populations, as indicated by the first visible active individual of the survey.

**Table 1.** White-clawed crayfish (*Austropotamobius pallipes*) ethogram. Adapted from Bergman and Moore (2003), Panksepp and Huber (2004) and McFarlane *et al.* (2019b)

Behaviour sub-group	Behaviour	Description
**Active**	Threat display	Crayfish rear up and spread chelae
	Displacing	Crayfish displace conspecifics from a refuge
	Attacking	Crayfish grasp or pull at conspecifics appendages with their chelae
	Non-agonistic interactions	Crayfish direct non-agonistic behaviour at nearby conspecific, e.g. exploratory behaviour with antennae
**Passive /Reactive**	Displaced	Crayfish retreating from a refuge when displaced by a conspecific
	Jack-knifing	Rapid backwards swimming propelled by tail and abdominal segments
	Retreating	Crayfish walks backwards with walking legs
	Attacked	Conspecific is grasping or pulling at crayfish’s appendages
**Non-interactive**	Resting	Any period of non-movement outside a refuge. Most appendages are inactive but may move periodically
	Grooming	Crayfish clean their antennae or carapace with appendages, chelipeds or mouthparts
	Foraging	Crayfish move first pair of walking legs from surrounding environment to mouthparts or manipulate external items with mouthparts, e.g. gravel
	Movement	Crayfish move forward or backward with no apparent behavioural motivator
	Refuge	Any period in which crayfish reside fully within a refuge.

### Data processing and statistical analysis

#### Data processing

Of the state behaviours observed, two were selected for further analysis. Although species-specific welfare indicators have yet to be validated, studies on a number of related species suggest that inappetence and variations in activity levels can be indicators of poor welfare (Narshi *et al.* 2022). For this reason, refuge use (which may indicate activity levels) and foraging were included. Recordings of displacing, attacking, and threat display were grouped as agonistic behaviour. Heightened agonistic behaviour could indicate disruption to social stability (Jiménez-Morales *et al.* 2018). If these behaviours were more prevalent in one population relative to another, or were more prevalent following a translocation event, they could therefore indicate compromised welfare.

#### Statistical analysis

All analyses and graph production were undertaken in RStudio (v2024.09.0+375; R Core Team 2025), running from R (4.5.1). Figures were generated using the ggplot package (Wickham *et al.* 2007) in RStudio. Proportions of each behaviour were calculated in base RStudio to generate activity budgets. Interobserver reliability was calculated using the cohen.kappa function (psych package) (Revelle 2019. Results of Cohen’s Kappa test consistently returned a result of > 0.8, indicating a high level of observer consistency (McHugh 2012). Differences in frequency of agonistic behaviour between populations (captive crayfish vs pre-introduction resident crayfish and pre-introduction resident crayfish vs post introduction resident crayfish) were determined using the Fisher’s exact test (fisher.test function) in RStudio. R’s generalised linear model (GLM) function (stats package) was used to assess the influence of several variables, including weather conditions, lunar phase, ambient temperature, time of night, date, predator presence, number of visible individuals, water chemistry, sex, and population on foraging and refuge duration. Refuge and foraging durations were created by adding the total duration for each individual behaviour in each individual survey. Although water chemistry measurements (NH_3_, NO_2_, NO_3_, Ca, PO_4_, pH and KH) were recorded throughout the surveying period, they showed little variation and remained within parameters of tolerable conditions for white-clawed crayfish (Haddaway *et al.* 2015). Moreover, they showed a high level of collinearity and affected the validity of model outputs when included. These factors were therefore excluded as predictor variables. As behavioural data for captive-born crayfish were limited post-introduction, pre-introduction data were selected for models comparing wild and captive crayfish behaviour. Individual sex groups (e.g. male resident, female captive) were not included in these models as these had aliased coefficients with the ‘population’ predictor variable. As these variables are highly related, population (captive or wild) was included to produce an overview of its effect on behaviour duration. We constructed the following models to assess influences on captive and wild crayfish behaviour:

- Foraging duration as response variable, with predictor variables population, date, time of night, ambient temperature, dissolved oxygen content, weather, lunar phase, number of visible individuals and predator presence; and
- Refuge duration as response variable, with predictor variables population, date, time of night, ambient temperature, dissolved oxygen content, weather, lunar phase, number of visible individuals and predator presence.

To analyse effects of translocation events on wild crayfish behaviour (as well as any other influential factors), separate models were constructed. Both pre- and post-introduction data were included in these models as wild crayfish were recorded consistently throughout both periods. We constructed the following additional models:

- Foraging duration as response variable, with predictor variables sex, date, time of night, ambient temperature, dissolved oxygen content, weather, lunar phase, number of visible individuals and predator presence; and
- Refuge duration as response variable, with predictor variables sex, date, time of night, ambient temperature, dissolved oxygen content, weather, lunar phase, number of visible individuals and predator presence.

Multicollinearity for each GLM was evaluated using variance inflation factors (VIF) from the car package (Fox *et al.* 2001) in RStudio; all values were below 10, indicating minimal collinearity between variables (Chaterjee & Hadi 2012). Residual diagnostics were also conducted using the base R plot function, showing no major violations of assumptions. Cook’s distance (cooks.distance function) was used to assess influential points, and no points were found to excessively influence any of the models in RStudio. Model fit was evaluated using akaike information criterion (AIC function) in RStudio.

## Results

### Body condition

Body condition scores of recaptured resident and translocated captive-reared crayfish remained consistent between the pre- and post-introduction period (Table 2).

**Table 2.** Body condition scores and carapace length of recaptured white-clawed crayfish (*Austropotamobius pallipes*) individuals before and after translocation event

Provenance	Tag no.	Sex	Pre-introduction size (mm)	Post-introduction size (mm)	Difference (mm)	Pre-introduction condition score	Post-introduction condition score
**Wild**	11916180	M	36.3	39.3	3	0	0
	11916141	M	36.4	39.1	2.7	2	2
	11916179	F	32.5	35.2	2.7	0	0
	11916132	F	32.8	33.7	0.9	2	2
	11916158	M	39.5	42.5	3	3	3
	11916165	M	40.5	42.5	2	1	1
	11916127	F	35	36	1	2	2
**Captive**	11916157	F	29.5	32.6	3.1	0	0
	11916140	M	30.5	33.4	2.9	0	0

### Differences in agonistic behaviour

Fisher’s exact test results indicated the frequency of agonistic behaviour in captive crayfish was not significantly different from wild crayfish (*P* = 0.32). However, frequency of agonistic behaviour was significantly higher (*P* < 0.001) in the wild population post-introduction, compared to pre-introduction, with 0.44 occurrences per session on average.

### Influences on captive and wild crayfish behaviour (pre-introduction)

Mean foraging duration was 306 (± 93.3) s in light rain and 286 (± 259) s in partly cloudy conditions, with both weather effects significantly reducing foraging duration (light rain: *P* = 0.0218; partly cloudy: *P* = 0.0100). No significant predictors on refuge duration were identified. Additional variables with no significant effect on behaviour durations can be seen in Table S2 (Supplementary material).

**Figure 3.**
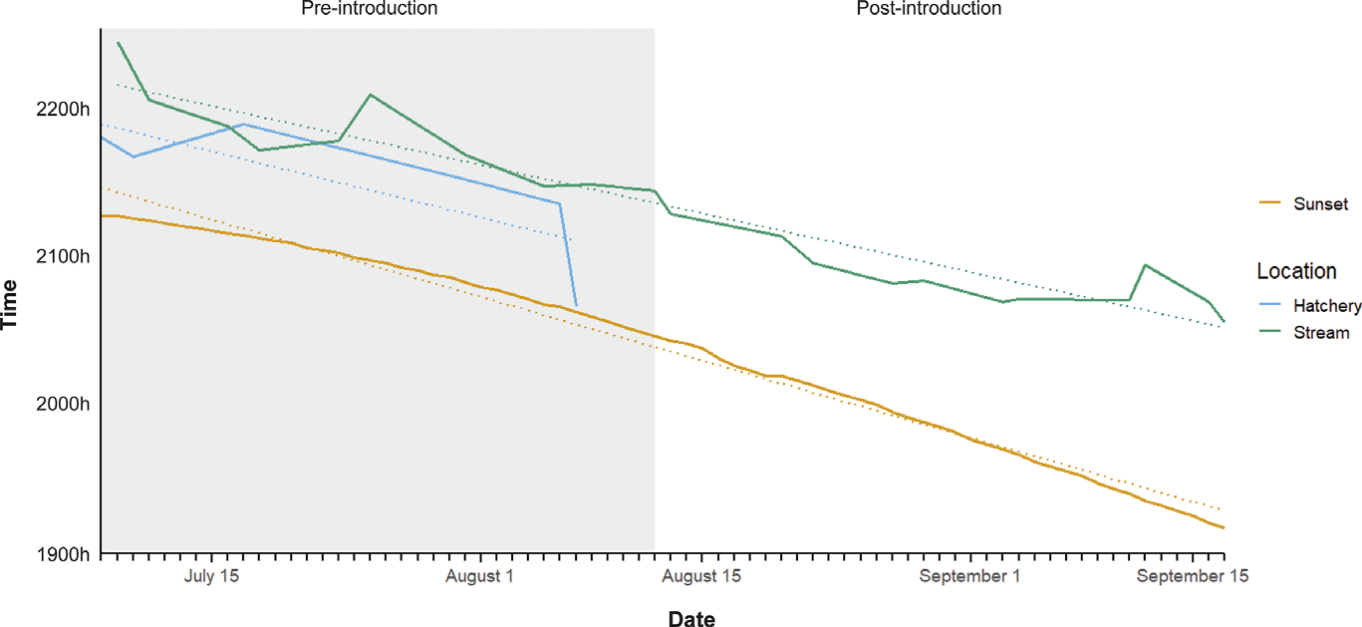
Emergence time of captive (hatchery) and resident (stream) white-clawed crayfish (*Austropotamobius pallipes*), determined by the first recorded active individual per survey. Dashed line depicts the line of best fit. Grey area represents pre-introduction period. Sunset time is provided as reference data.

**Figure 4.**
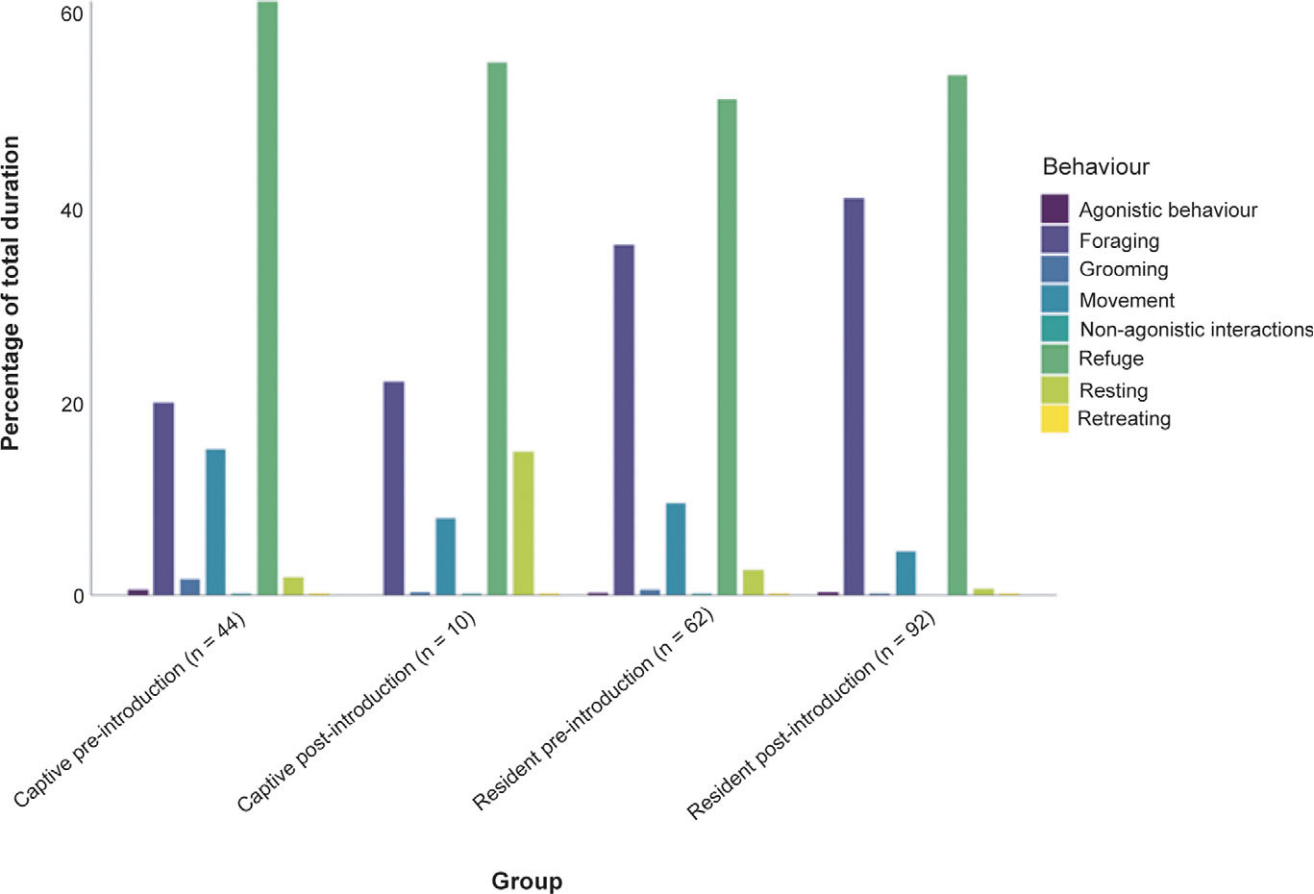
Activity budgets across captive-born and wild resident white-clawed crayfish (*Austropotamobius pallipes*), before and after introduction event. Key indicates behaviour category (agonistic behaviour = instances of threat display, attacking, displacing). N = number of surveys.

Emergence times showed a degree of variation between captive and wild individuals (Figure 3), with captive individuals emerging earlier, although these trends were based on limited survey periods pre-introduction (n = 16). Although behavioural surveys of post-introduction, captive-born crayfish were limited, and only pre-introduction data were assessed in linear models comparing captive and wild individuals, activity budgets varied between pre- and post-introduction periods for captive-born crayfish (Figure 4).

### Influences on wild crayfish behaviour (pre- and post-introduction)

**Figure 5.**
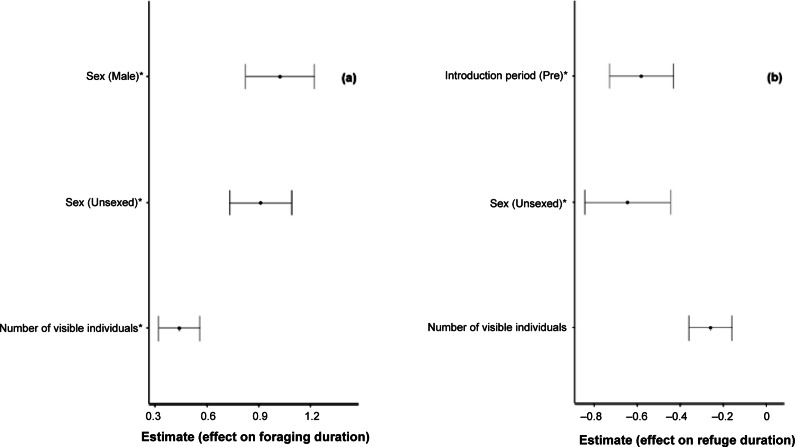
Significant predictors of foraging duration (a) and refuge duration (b) (wild white-clawed crayfish [*Austropotamobius pallipes*], pre- and post-introduction). Points represent the estimated effect sizes for each predictor, with horizontal bars indicating the 95% confidence intervals around these estimates. Sex had a significant positive effect on foraging duration, with Male and Unsexed individuals foraging longer compared with females. Higher numbers of visible individuals was also associated with longer foraging durations. Pre-introduction period had a significant negative effect on refuge duration, as did Sex (Unsexed) and number of visible individuals, each associated with shorter refuge durations. Only significant levels of categorical predictors are displayed; non-significant levels and variables are provided in Table S3 (see Supplementary material).

Individual sex groups were a significant predictor of foraging duration; male crayfish (β̂ = 1.0229 [± 0.20]; *P* < 0.001) and crayfish of unknown sex (β̂ = 0.9094 [± 0.18]; *P* < 0.001) exhibited longer foraging durations compared with females. Number of visible individuals was also a significant predictor, with higher numbers of visible crayfish associated with longer foraging durations (β̂ = 0.4392 [± 0.12]; *P* < 0.001). Other variables, including introduction period (pre or post) did not show a significant effect on foraging duration. For refuge duration, pre-introduction period (β̂ = –0.581 [± 0.15]; *P* = 0.0354) showed a significant negative effect on refuge duration, indicating refuge durations were lower during this period. The group of crayfish of unknown sex displayed significantly lower refuge use (β̂ = –0.645 [± 0.20]; *P* = 0.0093). As expected, refuge duration was significantly reduced when a higher number of individuals were visible in the stream (β̂ = –0.259 [± 0.10]; *P* = 0.0199). Significant predictors are recorded in Figure 5, and additional variables with no significant effect on behaviour durations can be seen in Table S3 (see Supplementary material).

## Discussion

### Implications of translocations

Comparison of captive and wild individuals, pre-introduction, highlighted no statistically significant differences in behaviour between populations, however due to the limitations of the study size, this is far from conclusive.

Differences were observed in the emergence times (i.e. the first instance of crayfish recorded exiting a refuge during surveys) of each population pre-introduction, with captive individuals tending to emerge earlier than the wild population, although this is based upon limited data. This could be considered a maladaptive behaviour as it may increase chances of predation in the wild, which in turn reduces the viability of a translocation. Reasons for this behavioural difference are likely associated with differences in environments. Many crayfish species are nocturnal, which is believed, in part, to be associated with predator avoidance (Davis & Huber 2007). In captivity, in the absence of predators, selection pressures are not acting upon individuals which would favour later emergence, and daytime feeding schedules may encourage activity earlier in the day. Similar behaviour has been observed in New Zealand rock lobsters (*Jasus edwardsii* Hutton 1875); however, after release, their emergence times aligned with those of wild populations as a response to heightened predator pressure (Oliver *et al.* 2006). Laboratory studies indicate that other crayfish species *Paranephrops zealandicus* (White 1874) and *Faxonius propinquus* (Girard 1852), modify their behaviour after exposure to predators (Stein & Magnusson 1976; Shave *et al.* 1994), which could suggest similar adaptability in introduced white-clawed crayfish.

Our results also suggest that introduction events may have an impact on resident crayfish. Refuge use was significantly higher post-introduction, which may be indicative of disturbance to the wild population. Although numbers of agonistic behaviours did not differ greatly between pre- and post-introduction periods, statistical analysis indicated an increase in the likelihood of agonistic interactions between these periods. It is possible that the introduction of new individuals into the population disrupted established social hierarchies, affecting their behaviour. Complex hierarchies exist in crayfish populations, which are established through agonistic interactions (Jiménez- Morales *et al.* 2018). When faced with novel individuals, these hierarchies are disrupted resulting in a higher frequency of agonistic behaviours. In response to agonistic interactions, subordinate crayfish have been shown to exhibit negative phototaxis (Bacqué-Cazenave *et al.* 2017), which may explain the increase in refuge use post-release. It should be noted that in this study, only six captive individuals were released into the resident population. Often supplementations involve the introduction of greater numbers of crayfish (Nightingale *et al.* 2017), which may amplify the impact of introductions on wild populations. Crayfish rely extensively upon chemical cues to understand their surroundings and establish social hierarchies (Horner *et al.* 2008) and moderate their behaviour based on these chemical signals (Schneider *et al.* 1999; Wood *et al.* 2018), meaning that even a small number of unfamiliar individuals has the potential to affect resident crayfish. Although we detected an increase in the likelihood of agonistic behaviour post-introduction, captive and resident crayfish showed no significant difference in the pre-introduction period. This is notable as studies comparing captive-born and wild individuals suggest captive animals display more aggressive behaviour (Kelley *et al.* 2006), although aggressive behaviour in captive populations may be affected by several factors including population density and resource availability. Increased aggressive behaviour in captive-born populations could have negative welfare implications in a translocation context, however this result was not seen here.

Despite the behavioural differences highlighted, comparison of the body condition of recaptured individuals pre- and post-introduction indicated no deterioration in condition. Captive-born individuals showed a comparable level of growth to resident individuals, suggesting they were able to successfully adapt to the novel environment. Although behavioural budgets of captive-born crayfish differed somewhat post-introduction, this may not be representative, as surveys of released individuals were limited.

### Other predictors of behaviour

Although differences were observed in certain behaviours between captive and wild individuals, as well as between resident crayfish pre- and post-introduction, much of the behavioural variation observed was not accounted for by population or the introduction event. Our results highlight several alternative explanations for crayfish behaviour. Weather was particularly influential on crayfish behaviour, with light rain and cloudy conditions associated with significantly reduced foraging time. We also observed during one night of heavy rain that captive crayfish were highly active and repeatedly jack-knifed to the surface, although this was not identified as a significant influence on behaviour durations, potentially due to a limited sample of observations under these conditions. To the authors’ knowledge, this is a potential predictor of white-clawed crayfish behaviour that has not been reported before.

Sex-based differences in behaviour were also observed; crayfish where sex was not determinable and male crayfish showed reduced refuge time and increased foraging time compared to females, post-introduction. It is possible that the majority of crayfish of indeterminable sex observed were male as their behaviour patterns mirrored that of sexed males. The increased foraging and reduced refuge time of males may be explained by higher activity levels in male crayfish and their propensity to move larger distances (Robinson *et al.* 2000), making them more visible in behaviours outside of the refuge, as well as the increased nutritional demand of males due to their larger body size, and higher growth rates (Nightingale *et al.* 2018b). Crayfish behavioural data were recorded from a population of mixed body size and age, but sampling limitations prevented the inclusion of these factors in analysis. Size and age-based characteristics could therefore provide alternate explanations of sex-based behavioural differences we observed.

Lunar phases have been shown to significantly influence activity levels in the crayfish species *Faxonius virilis* (Hagen 1870) (Mitchell & Hazlett 1996) and moulting cycles in *Astacus astacus* (Linnaeus 1758) (Franke & Hoerstgen-Schwark 2013). Our study highlighted no significant influence of lunar cycles on the behaviour of white-clawed crayfish. Anecdotally, increased levels of agonistic behaviour were observed on one night of surveying during the new moon. It is possible that lunar phase influenced this behaviour, as it has previously been reported that a related species, *F. virilis*, exhibited elevated activity levels during a new moon (Mitchell & Hazlett 1996). As this was not a primary focus for the study, the limited sampling under these conditions prevents drawing definitive conclusions regarding the effect of lunar phase on white-clawed crayfish behaviour.

Refuge use was significantly lower, and foraging durations significantly higher when greater numbers of wild crayfish were visible in the study area. These results may correspond to low or high activity periods in our study population. Alternatively, it could suggest that white-clawed crayfish behaviour is influenced by conspecifics, as seen in other decapod crustacean species (e.g. Van Maurik & Wortham 2014; Chak *et al.* 2015).

Time of day and year seemed to affect crayfish behaviour, suggesting activity budgets varied over the survey period. Although agonistic behaviour was more common in the post-introduction stage, seasonal behavioural changes may also provide an alternative explanation of our results. The post-introduction period approached the species’ reproductive season (as evidenced by recaptured females in glair [when developed, glair glands are visible on the ventral abdominal surface]), which has been associated with changes in agonistic behaviour (Villanelli & Gherardi 1998; Martin & Moore 2010). Additionally, although our analysis found evidence that crayfish behaviour was influenced by introduction period (pre or post), crayfish were captured and tagged prior to the behavioural survey period. Differences in behaviour over the course of the study period could therefore also be affected by the initial events of capture and tagging.

During surveying, some wild crayfish were observed using overhanging root systems to forage at and above the water’s surface. Previous studies demonstrate that crayfish are opportunistic feeders with a high degree of behavioural plasticity (Gherardi *et al.* 2004; Vesely *et al.* 2020). However, in captivity, the opportunity for surface level foraging is not available due to the risk of escape.

### Study limitations

During surveying, individuals were grouped by sex (or lack of determinable sex) to identify sex-based determinants of behaviour. However, this reduced our ability to identify influences on behaviour that occur at the individual level, which could be significant in determining the welfare needs of crayfish. Crayfish species exhibit personality traits (Galib *et al.* 2022) and these traits can be highly influential on responses to external stimuli such as predators or interspecific competitors (Vainikka *et al.* 2010). Factors such as body size and age also influence crayfish dominance hierarchies and behaviours (Kubec *et al.* 2019), but these factors were not recorded in our study. Moulting periods can also reduce foraging in crayfish species (Culley & Duobinis-Gray 1987) and were not accounted for in this study. Bristol Zoological Society housed several white-clawed crayfish populations that were not included in this study but were observed emerging earlier in the evening than the studied population. Our results may therefore not be representative of captive crayfish populations generally. Notably, all captive individuals we recorded were a similar age and size, in contrast with the wild population where these traits varied, so differences observed here could reflect differences in individual, size or age-based characteristics.

While efforts were made to monitor individual released crayfish following introduction by marking, the complexity of relocating individuals in a natural setting meant that they could not be consistently observed in the post-introduction period. Our study was also limited generally by the small sample size of captive crayfish recorded, which presents difficulties in generalising our findings to all captive crayfish. Moreover, the small number of crayfish within the hatchery increased the possibility of autocorrelation, and our results may not reflect the activity of crayfish housed in different conditions, such as higher stocking densities (Nightingale *et al.* 2018b). Observations of some behaviours, such as agonistic behaviour, were also limited meaning our ability to apply statistical analysis, such as generalised linear models, and test alternative explanations was restricted.

### Implications for practice

As crayfish species exhibit individual behavioural differences, future studies would benefit from following individual responses to translocation and including body size and age data in analyses to better isolate significant determinants of crayfish behaviour. Applying methods outlined here to larger data samples would further elucidate influences on white-clawed crayfish behaviour, as our study was limited by both small samples of captive study subjects and individual behaviours. Tracking movements of crayfish using telemetry equipment pre- and post-introduction would be beneficial in determining the effects of translocation on crayfish welfare, as well as aiding in location of individuals for behavioural surveying. We also found that the behavioural data used in our analysis had a range of interpretations, so we recommend research aimed at developing unambiguous welfare indicators for this species. Long-term monitoring would also provide a clearer picture of translocation impacts. Previous studies on related species indicate hierarchies stabilise over time (Goessmann *et al.* 2000) meaning behaviour recorded post-introduction may eventually revert to a pre-introduction state. Our results also indicate that stimuli such as weather, lunar phase, and terrestrial foraging areas may be important in the behaviour of white-clawed crayfish. Failure to replicate features of wild environments in captivity has the potential to compromise expression of natural behaviour, and lack of exposure to features of wild environments may reduce adaptability of released individuals to natural settings, negatively affecting welfare (Rabin 2003; Madden *et al.* 2020). We also found evidence that behaviour varied based on numbers of visible crayfish, which could form the basis of further studies on sociality in this species.

### Animal welfare implications

Our results provide some evidence that the translocation affected crayfish behaviour, which may indicate a negative impact on their welfare, but further studies are needed to clarify this. Despite the potential impact of the translocation on crayfish behaviour and welfare, their physical function did not seem to be affected, as we found that survival and growth rates of translocated captive-born crayfish mirrored those of their wild counterparts. Exposure to features of natural environments may allow expression of full behavioural repertoires, which could enhance welfare in both captive populations and captive-born crayfish introduced to natural settings. These features include weather and lunar cycles, as well as interaction with conspecifics and access to terrestrial feeding areas.

## Conclusion

Overall, our results provide some evidence that translocation events cause behavioural changes in wild crayfish, with captive-born and wild crayfish displaying behavioural differences, which could have negative welfare implications in a translocation context. However, healthy growth rates and body conditions were observed in both groups following introduction, and we propose that other factors may be important in behavioural regulation, such as weather conditions. We suggest that replicating features of natural environments could enhance captive-born crayfish welfare and increase the potential of successful translocation. We also recommend that further studies, which elucidate the long-term effects of introduction events on crayfish welfare, should look to further develop welfare indicators, and monitor welfare impacts on individual crayfish, as well as captive-born crayfish, post-introduction. To our knowledge, this study is unique in examining invertebrate welfare in a translocation context, indicating significant gaps in current research.

## Supporting information

10.1017/awf.2026.10080.sm001 Crowe et al. supplementary material Crowe et al. supplementary material

## Supplementary material

The supplementary material for this article can be found at http://doi.org/10.1017/awf.2026.10080.
