# Supplementary material for: Welfare considerations during a conservation translocation of white-clawed crayfish (Austropotamobius pallipes): Proof of concept
Source: Anim Welf. 2026 Apr 13;35:e27. doi: 10.1017/awf.2026.10080 (PMC13101029; doi:10.1017/awf.2026.10080)
Supplement: Crowe et al. supplementary material [file S0962728626100803sup001.pdf]

Welfare considerations during a conservation translocation of white-clawed crayfish (*Austropotamobius pallipes*): Proof of concept: Supplementary material

Imogen Crowe, Oliver Heley https://orcid.org/0009-0006-2787-3209, Alice Dancer, Ricardo Lemos de Figueiredo, Nicola Cook, Jen Nightingale, Sarah Richdon https://orcid.org/0000-0002-8923-2523

Bristol Zoological Society, Bristol, UK

Author for correspondence: Sarah Richdon, email: srichdon@gmail.com

Table S1: White-clawed crayfish (*Austropotamobius pallipes*) body condition scoring categories

Rostrum damage
Missing/damaged claw R
Missing/damaged claw L
Missing/damaged first leg R
Missing/damaged first leg L
Missing/damaged second leg R
Missing/damaged second leg L
Missing/damaged third leg R
Missing/damaged third leg L
Missing/damaged fourth leg R
Missing/damaged fourth leg L
Missing/damaged antenna R
Missing/damaged antenna L
Porcelain disease
Burn spot

Table S2. Variables and levels of categorical variables included in GLMs (wild and captive white-clawed crayfish [*Austropotamobius pallipes*], pre-translocation). Variables and levels approaching significance (*P* = 0.05–0.09) in bold, significant (*P* =<0.05) in bold with asterisk (*).

Variables	Levels (for categorical predictors)
**Population**	**Wild**<br>Captive
**Weather***	**Partly cloudy***<br>**Light rain***<br>Overcast<br>Clear night<br>Heavy rain
**Lunar phase**	Crescent<br>**Gibbous**
Date	-
**Time**	-
Temperature	-
Dissolved oxygen content	-
**Predator presence**	-
Number of visible individuals	-

Table S3. Variables and levels of categorical variables included in GLMs (resident white-clawed crayfish [*Austropotamobius pallipes*], pre- and post-translocation). Variables and levels approaching significance (P = 0.05 – 0.09) in bold, significant (P = <0.05) bold with asterisk (*).

Variables	Levels (for categorical predictors)
**Sex***	**Unsexed***<br>**Male***<br>Female
**Weather**	**Overcast**<br>Clear night<br>Partly cloudy<br>Light rain
**Introduction (Translocation period)***	**Pre***<br>Post
Lunar phase	Crescent<br>Gibbous<br>Quarter<br>New moon<br>Full moon
**Date**	-
Time	-
Temperature	-
Dissolved oxygen content	-
Predator presence	-
**Number of visible individuals***	-
